# The Meaning of Chronic Disease Management in the Patient’s Environment: A Critical Ethnographic Study

**DOI:** 10.3390/healthcare14070882

**Published:** 2026-03-30

**Authors:** Valérie Loizeau, Rita Georges Nohra, Dominique Pougheon-Bertrand, Monique Rothan-Tondeur

**Affiliations:** 1 Assistance Publique Hôpitaux de Paris (AP-HP), Nursing Sciences Research Chair, F-75005 Paris, France; rothan-tondeur@univ-paris13.fr; 2 Chaire Recherche Sciences Infirmière, LEPS, UR 3412, UFR SMBH, Université Sorbonne Paris Nord, F-93430, Villetaneuse, France; rita.nohra@u-paris.fr; 3 Département Universitaire des Sciences Infirmières, Université Paris Cité, ECEVE, UMR 1123, Inserm, F-75010 Paris, France; 4 LEPS, UR 3412,UFR SMBH, Université Sorbonne Paris Nord, F-93430 Villetaneuse, France; dominique.pougheon-bertrand@univ-paris13.fr

**Keywords:** chronic disease, environment, ethnography, nursing support

## Abstract

**Highlights:**

**What are the main findings?**

- Patients need to feel able to express themselves and be heard, and this requires genuine listening to promote a sense of security.
- Encourage autonomy and person-centred leadership through digital medicine.

**What are the implications of the main findings?**

- Encouraging true autonomy based on people’s needs and desires through co-coaching allows us to follow the objectives of the Ottawa Charter
- Creating spaces for discussion between patients and staff, with genuine listening that fosters a sense of security and promotes person-centred leadership

**Abstract:**

Background The social, physical, and relational environment plays a particularly key role for people living with chronic illness. The available resources must match the needs and abilities of those individuals. Objectives This study aims to describe how people perceive their environment in relation to managing their chronic illness daily. Methods Ethnography was employed to collect and analyze data. The researcher visited each participant at home three times, making observations and conducting interviews. Results Fifteen people with cardiovascular disease took part in the study. Four themes emerged relating to their environment: self-expression/being listened to; decision-making/action; creating a safe space; and overcoming illness. Conclusions A supportive environment enables individuals to recognize their achievements based on the meaning they attribute to them. Although people adapt to their environment according to their abilities and needs, effective communication between people with chronic diseases and healthcare professionals remains essential.

## 1. Introduction

Chronic diseases are responsible for three out of five deaths worldwide and are attributed to four diseases: heart disease, lung disease, cancer, and diabetes [1]. This global epidemic poses a challenge for all countries, as the disability caused by chronic diseases and the ageing population requires effective management policies [2,3]. The impact of chronic diseases on a person’s life depends on the disease, its stage of development, and factors such as self-confidence, self-image, and recognition by others [4,5]. To enable individuals to cope, the means of encouraging them within a care relationship emphasize empowerment and expertise in the experience of the disease [6]. A person’s perception of their quality of life is based on self-assessment, which depends on internal and external factors [7,8]. The concept of health-related quality of life is also a key indicator in disease monitoring [9]. Quality of life appears to be linked to the environment in which a person lives. It is often associated with noise and air quality [10,11]. However, the environment also has connotations that include individual factors (age, disability, illness, and gender) and disparities related to social context [12,13].

**Figure 1:**
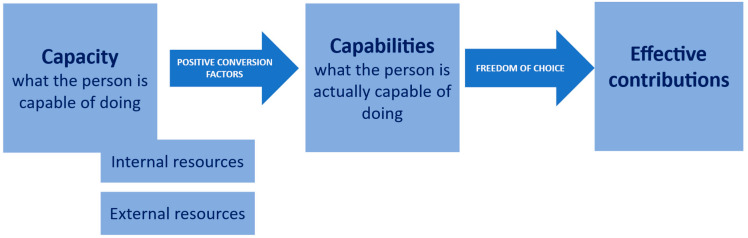
Illustration of the capability approach.

The design of an appropriate environment is essential to enable action, but it must bring together favourable conditions through the resources available [14]. An approach focused on empowerment looks at the characteristics of the environment as well as the opportunities available and people’s ability to exercise this power [15]. This environment, described by some authors as an enabling environment, was proposed in 2005 in ergonomics [16,17]. To describe it, Falzon draws on the capabilities approach [12] (Figure 1).

An enabling environment is an environment that enables capabilities and access to empowerment. An enabling environment is one that fosters capabilities, thereby facilitating access to the power to act. Furthermore, the capabilities approach supports individuals by focusing on their strengths and rights, enabling them to seize opportunities to act. The enabling environment is a key factor in individual empowerment. Moreover, a culture that empowers individuals to make their own decisions is crucial. [18]. Thus, the capability approach supports people by focusing on their strengths and rights to act [19]. The enabling environment is an ‘active ingredient’ of empowerment [20].

An environment that allows individuals to make their own decisions and develop their capacity for action is also shaped by culture. Culture refers to the lifestyles of individuals or groups in relation to values, beliefs, norms, and practices that are learned, shared, and passed on. In the field of nursing, concern for the environment has a long history and is relevant in all countries. Furthermore, the individual, health, and the environment are the foundations of the nursing discipline. Its omnipresence in care thinking does not guarantee its concrete implementation by professionals to ensure quality of life and the possibility of acting according to one’s choices.

Current literature considers the environments of people living with chronic diseases in relation to noise, pollution, and care structures. Similarly, quality of life is considered in relation to the person’s environment, albeit in a general way. To our knowledge, the living environment as perceived by the individual has not been considered.

This study uses a method that reflects the reality of people living with their illness in their daily lives, as well as their own frame of reference for developing their environment. This environmental context encompasses the events and life experiences that the individual interprets and uses to inform their decision-making processes. A critical ethnography approach was chosen in view of the population in question being vulnerable individuals and the power relations that may exist within the socio-cultural system. This variant of traditional ethnography enabled us to observe the present situation and imagine potential future scenarios, particularly stimulating the participants’ ability to act.

The aim of this study is to describe the meaning that people give to their environment in relation to the management of disease in their daily lives by directly observing their living environment.

## 2. Materials and Methods

This qualitative ethnographic study examines human behaviours and the notion of culture. An orientation towards critical ethnography [21,22] was chosen due to the selected population and power relations in the socio-cultural system [23]. In this study, the choice of this method stems from the reality of the individual living with their illness in their daily life and from observing their own frame of reference as they shape their environment. This environmental context concerns events and life experiences to which the individual attributes meaning, enabling them to guide their decisions. Data were collected using triangulation methods: observations, informal conversations, photographs, and interviews [24]. Data collection took place between July and December 2022.

### 2.1. Participants and Sampling

The study subjects were people living with chronic disease, the world’s leading cause of death [25]. The participants exhibited a range of cardiovascular disease characteristics, from myocardial infarction to intracardiac conduction problems and varying degrees of heart failure. The sampling was non-probability and purposive [26]. Leininger explains that 4–6 key individuals are sufficient to ‘obtain the necessary depth of data’ and allow the rigour of the ethnographic method to be maintained [23]. However, this study followed the rule in qualitative research regarding data saturation [27]. The selection was made with cardiology professionals, focusing on those meeting the inclusion criteria. Study investigators contacted participants by phone.

### 2.2. Data Collection Procedures

Two methods were used to collect data: observations and interviews. Situational observation is described as “a data collection tool in which the researcher becomes a witness to behaviours and practices by remaining in the places where they take place” [28]. The aim was to collect data on how people manage their environment [29]. A flexible interview guide (File S1 in Supplementary Materials) was used, based on the integrative literature review on supportive environments for people with chronic illnesses [30]. Each person received three visits from the researcher. The first two visits consisted of non-participatory observations and informal interviews with the individuals. During the third visit, a semi-structured interview was conducted. The interview guide was tested with two individuals to determine the degree of comprehension of the questions.

### 2.3. Data Analysis

The data were integrated, processed, and analyzed using logiciel ATLAS.ti (version 9; ATLAS.ti Scientific Software Development GmbH, Berlin, Allemang, Germany). Codes were assigned after reading the data. These codes came from the verbatim and observational sections. Each participant was also given space to tell their own story.

A four-phase analysis was then carried out according to the method proposed by Spradley [31]. This method highlights the significance of a person’s behaviour. These phases identify the most salient themes, which give meaning to people’s experiences of illness in their environment.

The analysis was validated by a second researcher from the Nursing Research Chair. Regular meetings were held with a third researcher to discuss the results.

The protocol was submitted to the university’s research and ethics committee and was accepted on 4 May 2021 (IRB No.: 00012021-25).

## 3. Results

The study included 15 people. After an initial analysis, 629 citations and 120 codes were developed on Atlas TI. These data were then analyzed to support people’s reports on their environment.

### 3.1. Socio-Demographic Characteristics

The participants in the study ranged in age from 26 to 77 years, with men and women similarly represented. Half of the people were employed; the others retired or were disabled. Half lived in flats, and the others in detached houses (Table 1).

### 3.2. Summary of Participants’ Experiences

This summary is written in the form of a short story to capture the participants’ everyday life experience. The goal was to tell people’s stories by connecting them to their empowerment. Two stories are confidential and anonymous. Two iconic stories were selected:

Experimental Patient 1 is a 56-year-old computer engineer, married with four children. He lives in a multi-story house in the city centre. He was diagnosed with the disease at a young age and said, “It hit me in the middle of my race.” Currently working from home, his space is on the table in the main room. Behind him and on the floor are all his medical records, as well as care and monitoring equipment and treatments. “My wife is unable to manage anything; I don’t ask her.” He interacts with the doctors and takes a stand, “I have questions, I need you to help me understand,” while doing research on the internet. Doctors and nurses do not respond to his emotions. “I was in need, I told the cardiologist that with the amount of medication I was taking, I was becoming depressed. The doctor sent him “to the shrink who prescribed me medication.” For him, it is paradoxical, he stops taking medication and goes to the psychiatrist, “no one has a solution to offer me”. When he is not well, he locks himself up, tells his wife that it will pass, and his wife waits for him. He concludes, “When I’m not well, I sleep”. He says, “I’ve argued with doctors before because the infantilizing aspect is unbearable. The doctors are the ‘knowers’ but I am involved in the choice”.

Experimental patient 2, 40 years old, currently on disability, former bakery salesperson. She lives with the youngest of her three children in an apartment in a small town with public transportation. Her heart disease is a difficult event to live through on every anniversary, “it stays with me, it’s part of me”, so she got a tattoo commemorating the event. Her family lives far away, but she often calls her mother. “My mom is my shrink”. She goes out a little bit, but because she lives on the third floor and there’s no elevator, she organizes her errands with her partner, her daughters, and her neighbor. She doesn’t really have a network of friends. During consultations, her doctor uses certain words, but she doesn’t dare say “I didn’t understand”. For example, he used the word “after-effects” and “I didn’t ask him to repeat it with simpler words”. She does what the doctor tells her to do because it is her heart. She looks at social networks and the internet, but never in relation to her illness. She takes her medication every morning, “it has become a ritual”. In the late afternoon, she is very tired, despite taking a nap, and those around her find it difficult to understand her.

The themes that describe the meaning people give to their environment in relation to illness are derived from the four-stage analysis proposed by Spradley [31], who identified four themes, each associated with sub-themes:

Theme 1: Being able to express oneself, to be heard: a matter of course sometimes … often ignored.

Managing emotions: a taboo subject, difficult to explore: It is a difficult subject for professionals and people alike. He keeps everything to himself, even to his wife (although he calls her “my head nurse ”), he always answers, “I am fine” (P7–117:6).

The doctor saved me, but what about afterwards? She says straight away “Doctor … is my savior” (P5—101:1). People are shocked by the violent entry of the disease into their lives; some speak of post-traumatic stress. They feel supported during the hospitalization phase, but not necessarily the support they would like.

Being at ease with their doctors, but talking about what? People said they could easily go to the doctor and ask questions (often prepared in advance). Some felt the doctor was not interested in their doubts. “I have low morale, I think it is due to my illness, I don’t talk to the doctor about it, he doesn’t ask me anything” (P15–158:3).

The entourage: support or not?

Some people involve their entourage, surround themselves with a trusted person, others not at all. We note that the men in a couple rely on their wives, “My wife knows how to deal with the disease if there is a problem” (P2–88:12), except for one patient we met, and for the singles, it is rather the children who serve as support.

Theme 2: The power to decide and act: a right that is not always simple in practice.

Deciding for oneself, according to one’s desires and environment, can be difficult. People living with a chronic disease fit into a proposed care scheme, some disciplined, “I do what the doctor tells me”, without really asking questions, others refusing to be passive, who say that one must “exercise one’s critical mind”. Finally, some wanted to give their opinion but ultimately let the doctor decide, “I trust him”, “he knows”.

Taking a stand and deciding, with or without the agreement of professionals: some people manage by consulting specialized organizations but leave the decision to the doctor. Mr. X initially refuses the transplant (despite the doctors’ insistence) “I’m tired, it’s been 5 years, I’m fed up”, then tells me that he finally accepts, and goes back on his decision for his family (P7–111:9). “I am obedient, rigorous in her treatments, her diet, her physical activity, even if she does not agree with the decisions, she trusts them”. (P11–132:11).

Opposing a decision and assuming the consequences alone: “The arrhythmologist sold me the idea that I could die suddenly, I needed time to make my decision. She refused the device because she couldn’t swim, they didn’t ask her how she was living. The doctor who did the ultrasound seemed to agree with me, my husband trusts me, he let me make this decision (although he is a doctor). On leaving the hospital the report said, “refused the device”, so he is following me” (P14–146:6). There is a relationship of submission between patient and doctor, and doctor and social network. Consent and opposition can complicate things.

Patient organizations: an unfamiliar environment: There is little communication on the subject; you must look for information yourself, but people do not feel the need to do so. “I’ll feel like I’m brooding, I don’t want to lock myself up in the disease” (P11–132:14). “The danger would be to stay locked up” (P1–84:4).

Theme 3: Being able to arrange a safe space: constrained and unequal freedom.

The creation of a safe space seems to be a common point for all the people we met, as safety allows them to manage the disease daily, to adapt, and to create links with their environment.

Learning or applying? Taking ownership of the disease: People learn in different ways: with professionals, during consultations, on forums, or by listening to their body. “I exchange a lot with my cardiologist to learn, my doctor is one of the important things, I tell him: I have questions, you have to help me understand” (P1–82:15). When people need to go into more detail, they use the internet but stay on general notions, for example, defibrillator and driving licence, medicines. “In rehabilitation I learned about meals, physical activity. I applied the first few times and then without a coach it is difficult to continue” (P2–89:17).” I would have liked a real exit interview, real coaching, someone to explain things to me” (P11–134:4).

Information and communication technologies (ICT): should they be tamed? In the end, 13 out of 15 people explained that they used it as a source of information about their illness. “I looked up triple bypass on the internet; it saved my life once” (P6–107:5). Social networks are also a means of communication and a place for sharing outside of the disease. Some people use connected objects to monitor and secure themselves. He sets his connected watch to 130 maxima, monitors his heart rate when he walks, and rests when he feels better. (P8–117:20)

Medication: an unchanging ritual in the daily life of a sick person. Medication is common to all participants. Only four people (three women and one young person) do not prepare their treatment themselves.

Being able to arrange one’s living space: a constrained and unequal freedom. The people we met lived in various places and had different housing. People adapt, and none changed their living space after their episode. People adapt their space according to needs. There is a notion of degrees of autonomy relating to old age or illness.

Theme 4: Breaking out of the vicious circle of illness: a desire to build.

Getting out of the disease by interacting with the environment: She does not feel sick, does not feel that she has a chronic illness. She says that it is a very strong life/couple experience. “Now I know what is the priority in life” (P11–131:10). “You have to deal with it, you have to live with it, it’s a knitting” (P14–125:8).

Stop everything after the accident: start again, but how? After the first heart attack, he returned to work as normal, with a lot of stress. Then, the second time, the manager asked him to work part-time, but in the end, “they stop you all of a sudden and I am useless” (P7–111:14) “My illness prevented me from returning to work in France, then I worked with disabled children but they fired me because I had to work part-time” (P2–88:1).

With or without family? Social isolation is sometimes desired and sometimes experienced. When experienced, fatigue is often a common factor that requires adaptation. Some people want interaction to think about something other than illness. “Friends, we don’t see them anymore, they are in the same state, we have memories. We communicate with them by phone and email, when we phone each other, it means we are in good shape” (P10–127:5).

**Figure 2:**
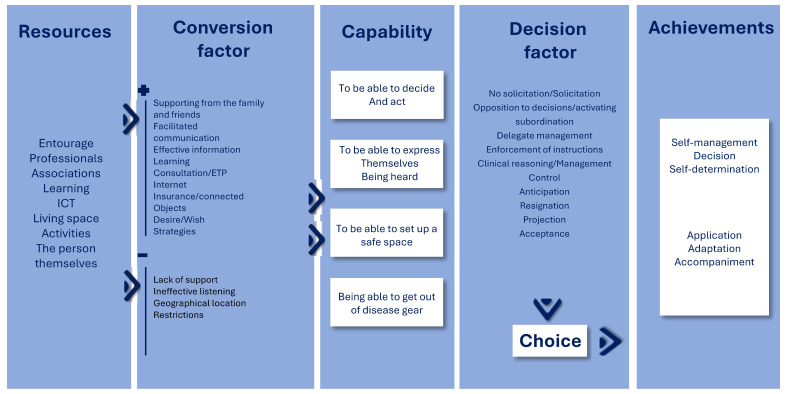
The capability approach in chronic illness.

The enabling environment in heart disease:

People describe their environment through the meaning they attribute to it. Resources include friends, professionals, a place to live, learning about the disease, and the self. Resources alone are insufficient to create a favourable environment and realize the person’s capacities, so conversion factors are needed, which can be classified as positive and negative. This study shows that both types of factors lead to the development of capabilities, such as deciding and acting, expressing oneself and being heard, providing a safe space, and moving away from the disease. The last part led to achievements and highlighted behaviours: some self-manage; others adapt in a more constrained style. However, both make choices. The following diagram shows the five parts of the support process (Figure 2).

## 4. Discussion

### 4.1. Discussion of the Method

Critical ethnography allowed the researchers to voice their feelings about care. Participants and researchers appreciated this. The study’s limitations stem from the subjective analysis of results by the researcher. A second researcher validated the process, while a third supervised.

### 4.2. Discussion of the Results

This research examined how people contribute to environmental development and its potential facilitative role. It built on prior studies, highlighting the link between active participation in care and better illness self-management. Our research focused on the quality of participation rather than self-management effectiveness. Analysis supported Sen’s capabilities definition in relation to daily life illness management.

Sedlar’s study revealed negative emotions like helplessness, hopelessness, and worthlessness for two out of three patients. Participants struggled to discuss their emotions with healthcare professionals [32]. Some countries have implemented strategies to improve communication, such as the ‘Matron effect’ [33] and ‘case management’ [34]. This support allows for coordination of follow-up services and responses to individuals’ needs.

When discussing democracy in health, patient associations are rarely referenced. Only three people mentioned them. People do not want to join associations to get answers, fight isolation, or exchange information, even though they can lead to personal transformation [4].

Ploeg’s study emphasizes the role of family and friends in informal support. Some people in our study relied only on themselves [35]. Dunbar discusses how family influences self-management, especially material aspects of daily life. People rely on others for support. Those we met chose not to ask for help to avoid overwhelm. Self-management and choice are often secret, and patients may be reluctant to talk about their condition [32].

Being able to decide and act:

The concept of vulnerability due to the disease necessitates shared decision-making, driven by the expertise of healthcare professionals and individuals. Complex decision-making involving choices requires individualized interviews. In our study, people were given two choices: (1) to let the doctor decide, as they believe doctors have expertise, or (2) to seek information independently. Individuals are also influenced by their doctors’ attitudes. Ploeg’s study shows some doctors focus on their patients’ goals, not what the interviewees want [35]. Beyond this, the two individuals likely made decisions based on what Reach developed in reactance theory, triggering critical thinking when faced with freedom threats [36].

Be able to create a safe space:

A safe environment that allows people to manage their disease is established through daily learning. People develop experiential knowledge [37] that allows them to analyze and reason clinically. In this study, they acquired this knowledge directly from doctors. However, people also develop strategies if they do not understand everything, such as looking up the effects of the disease on the internet.

The appropriation of people’s living space to make it a safe place varies according to their social context. Nevertheless, people have an emotional attachment to their living space and adapt it to the constraints of the disease as they adapt themselves [38]. At this stage, health professionals could intervene more widely in certain situations using their knowledge of resources, place of residence, and social support [39].

Breaking out of the illness trap:

Illness causes necessary adaptations. Some allow for negotiation, e.g., moving home, a new job, or social benefits [40]. Others involve distancing from the illness. This can be about setting up a respite, which can be outside the family and social circle.

ICTs have been used little in the management of the disease, but are used by the elderly when mobilization becomes more complicated. This is in line with Allida’s study. Technologies are increasingly used in the world, but not systematically considered at the health level [41]. Using Fernagu’s schema of capabilities, we highlight a difference in chronic diseases: negative conversion factors can enable capabilities. There are also interactions between opportunities related to resources and people’s freedom that allow power to act for them [18].

People succeed through their choices. One person refused a medical device and now takes medication. Another person swims in the ocean every year. A surgeon followed his instincts and is retraining for a new career.

### 4.3. Limitations

These are related to the analysis of the results, which is subject to the researcher’s subjective opinion. To overcome this, a second researcher validated the four-phase process, and a third researcher supervised it.

Similarly, the study focused on a population of people living with either heart disease or heart disease associated with diabetes, a consequence often found in people’s health histories. One might wonder whether the behaviours of these two populations in managing the disease could be associated, but we focused on the individual’s environment. In his scoping review, Riegel discusses siloed care by specialty, which hinders the transfer of information and the sharing of knowledge between scientific communities.

## 5. Conclusions

The enabling environment, which allows access to achievements through capabilities, was described in terms of the meaning people attribute to it. Ultimately, individuals adapt according to their capabilities and desires. We found that some people make decisions themselves, while others rely on professionals. Even if they are constrained by an inadequate system, they develop the capacity to adapt. Furthermore, improving communication between individuals with chronic diseases and professionals appears to be a key area for future development.

## Figures and Tables

**Table 1:** Characteristics of participants.

Characteristics of Participants	*n* = 15
Age (mean and standard deviation)	55.8 (14.4)
Female	8 (53%)
Male	7 (47%)
Marital status	
Married	9 (60%)
Divorced	4 (26%)
Single	1 (7%)
Widowed	1 (7%)
Level of education	
Vocational studies	8 (53%)
Baccalaureate	1 (7%)
Advanced technician’s certificate	1 (7%)
Bachelor’s degree	3 (19%)
Master’s degree	2 (14%)
Administrative position	
Activity	7 (47%)
Unemployment	1 (7%)
Disability	1 (7%)
Retired	6 (40%)
Place of residence	
House	8 (53%)
Apartment	7 (47%)

## Data Availability

The authors are available to provide specific information from the data collections.

## Supplementary Materials

The following supporting information can be downloaded at: https://www.mdpi.com/article/10.3390/healthcare14070882/s1, File S1: Interview guide.
