# Supplementary material for: The Meaning of Chronic Disease Management in the Patient’s Environment: A Critical Ethnographic Study
Source: Healthcare (Basel). 2026 Mar 30;14(7):882. doi: 10.3390/healthcare14070882 (PMC13073744; doi:10.3390/healthcare14070882)
Supplement: Supplementary file 1 [file healthcare-14-00882-s001.zip › healthcare-4085403-supplementary.pdf]

Interview guide

Question 1

What is your daily routine when it comes to dealing with your illness?

(Care, learning, professionals, family and friends, organization)

Question 2

Can you tell me about your collaboration with healthcare professionals: doctors, nurses, other professionals?

(Concept of trust, roles of each person, decision-making, frequency of meetings, availability)

Question 3

How do you ensure that your expectations, needs, and concerns are taken into account?

(Position of demand, expectation, power)

Question 4

Can you tell me about your support network for coping with the disease? (Healthcare professionals, family, friends, support group, peers, association)

Question 5

Ideally, what would you like to see to improve your care? What would your dream environment be like?
